# The Association between Sleep Duration and 25-Hydroxyvitamin D Concentration with Obesity in an Elderly Korean Population: A Cross-Sectional Study

**DOI:** 10.3390/nu10050575

**Published:** 2018-05-08

**Authors:** Miae Doo

**Affiliations:** Department of Food and Nutrition, Kunsan National University, Daehak-ro 558, Kunsan, Jeonbuk 54150, Korea; miae_doo@kunsan.ac.kr; Tel.: +82-63-469-4635; Fax: +82-63-466-2085

**Keywords:** 25-hydroxyvitamin D, dietary consumption, Korean National Health and Nutrition Examination Survey, sleep duration, obesity

## Abstract

Studies have recently reported an association between sleep duration and obesity in some individuals. Vitamin D deficiency is common in elderly populations and is also associated with obesity. In this study, the combined interaction effect of vitamin D levels and sleep duration on obesity-related variables was analyzed in 3757 individuals from an elderly Korean population using the Korean National Health and Nutrition Examination Survey. Significant differences were observed in the interaction effect of the vitamin D levels and sleep duration on obesity-related variables, including BMI (*p* = 0.004) and the risk of obesity (*p* < 0.001). Using vitamin D sufficient status and proper sleep duration as a reference, subjects with sufficient vitamin D did not differ in their risk of obesity regardless of their sleep duration. However, the risk of obesity tended to increase with short sleep duration rather than proper sleep duration among subjects who were vitamin D insufficient odds ratio (OR) (95% CI) = 1.293 (1.10–1.657) for proper sleep duration vs. 1.374 (1.066–1.770) for short sleep duration). Only the participants in the vitamin D deficient population who consumed less protein showed an increasing trend in the risk of obesity according to the sleep duration (OR (95% CI) = 1.645 (1.155–2.344) for proper sleep duration and 1.668 (1.156–2.406) for short sleep duration).

## 1. Introduction

Vitamin D is a fat-soluble vitamin, and its two main types are vitamin D2 (ergocalciferol) and vitamin D3 (cholecalciferol). Although vitamin D is mainly synthesized by dermal synthesis upon sunlight or ultraviolet light exposure, its synthesis could be limited under certain conditions, such as seasonal variations, older age, darker skin, and the use of sunscreen and protective clothing [1]. Dietary vitamin D is obtained from nutritional sources, including fatty fish, organ meats, fortified milks and margarines, egg yolks, mushrooms and supplements [2].

Vitamin D deficiency is a very common public health problem in elderly populations [3,4]. Due to aging-associated physical changes, such as a decrease in taste sensitivity, poor oral health, physical difficulties, and financial limitations [5], poor nutritional status and malnutrition are common in the older adult population [6]. These conditions are associated with dietary vitamin D deficiency [7,8]. Indeed, vitamin D deficiency has been reported to be associated with the prevalence of obesity [9,10,11]. According to a recent meta-analysis study, the prevalence of vitamin D deficiency was higher in overweight and obese individuals [10]. Additionally, obesity among the elderly is as prevalent as in younger and middle-aged populations globally [12]. Among Koreans older than 65 years, the prevalence of obesity is reported to be 32.6% and 42.8% for men and women, respectively [13]. Additionally, obesity is associated with a higher relative risk of mortality from all causes of chronic diseases and is influenced by different factors including lifestyle (dietary consumption, physical activity, and sleep pattern) and socioeconomic factors (gender, educational level, and household income).

The duration of daily sleep required is known to decrease with age [14]. However, in a previous study, elderly individuals reported that their average sleep duration was similar to or longer than that reported by younger and middle-aged individuals [15]. Nevertheless, in this study, older individuals slept for a shorter duration [16], and many older individuals complained of sleep problems, such as insomnia and poor sleep quality [17,18]. The association between poor sleep patterns and obesity has been reported [19,20,21]. In recent decades, sleep duration has been shown to decline, and a dramatic increase in the prevalence of obesity, which is linked to short sleep duration, has been observed during the same period [19]. In our previous studies involving Korean women, shorter sleep durations were associated with an increased risk of obesity [20]. In addition, differences in dietary macronutrient consumption are affected by sleep duration, which could potentially increase the risk of obesity. Several studies have reported significant associations between poor sleep quality and obesity in the elderly [21,22].

Therefore, I hypothesized that the combined interaction effect of vitamin D and sleep duration on obesity was caused by dietary macronutrient consumption among elderly populations. Therefore, the purpose of this study was to examine the combined interaction effect of 25-hydroxyvitamin D (25 (OH) D) and sleep duration on obesity-related variables among the elderly populations who participated in the Korean National Health and Nutrition Examination Survey (KNHANES). Moreover, this study examined how these interactions are affected by dietary macronutrient consumption.

## 2. Subjects and Methods

### 2.1. Study Design and Participant Selection

In this study, I used data from the KNHANES V (2010–2012) and VI (2013–2014), which are national representative, cross-sectional surveys that have been conducted annually by the Korean Centers for Disease Control and Prevention (KCDC) since 1998 [13]. The KNHANES consisted health interviews, health examinations, and dietary surveys to investigate the relationship between health and nutritional status. The participants in the KNHANES were selected using a complex, stratified multi-stage probability clustered sampling design among non-institutionalized civilians from the South Korean population. From a total of 41,102 participants (18,646 men and 22,456 women) in KNHANES V-VI (2010–2014), 3757 participants (1694 men and 2063 women) aged ≥65 years with a plausible daily energy consumption and no missing or inadequate data were enrolled in this study. All participants provided written informed consent, and the survey protocol was approved by the KCDC Institutional Review Board (IRB No. 2010-02CON-21-C, 2011-02CON-06-C, 2012-01EXP-01-2C, 2013-07OCN-03-4C, and 2013-12EXP-03-5C).

### 2.2. Data Collection

The general characteristics, anthropometric and blood biochemical variables, and dietary consumption data were collected from the KNHANES data. Self-reported data regarding general characteristics, such as gender, age, education level, subjective stress level, alcohol consumption, smoking status, physical activity, sleep duration, and dietary macronutrient consumption, were collected during health interviews. The anthropometric and blood biochemical data were measured during the health examinations.

The participants’ gender, age, education level, subjective stress level, alcohol consumption, smoking status, dietary supplement use, physical activity, and sleep duration were assessed via questionnaires. The educational levels were classified as “≤middle school” or “≥high school”. The living together was classified as “no”, with “unmarried, divorced, bereavement” and “yes”, with “married or remarried”. The level of subjective stress was measured using a 5-point Likert scale, and the participants were divided into “low” and “high” stress using a score of three as the cutoff value. The participants were classified as “non-drinkers” or “alcohol drinkers” based on their regular alcohol consumption and “non-smokers” or “current smokers” based on their current smoking habits. The use of dietary supplement was classified as “no” or “yes” during the prior year. Regular physical activity was classified as “no” or “yes” during the prior month. Sleep duration was assessed as the average number of sleeping hours per day, and was divided into <7.0 h a day and ≥7.0 h a day. A usual sleep duration of 7.0 h was used as a reference in accordance with previous studies [14,23].

Dietary consumption was assessed by dietitians using a food frequency questionnaire (FFQ) developed and validated for the KNHANES [24]. The FFQ consisted of 63 food items and 10 categories for the consumption frequency in 2010–2011 and 112 food items and 9 categories for the consumption frequency in 2012–2014, and these were used to calculate the twenty nutrients. The consumption of dietary macronutrients was calculated as a percentage of the total energy consumption. The percentage of total daily energy from proteins, fats, and carbohydrates (CHO) was divided by the median levels as follows: proteins: 12.50%, fats: 13.24%, and CHO: 72.90% for men and proteins: 11.84%, fats: 8.96%, and CHO: 78.76% for women.

The anthropometric variables, including height, body weight, and waist circumference (WC), were measured using standardized procedures. The body mass index (BMI) was calculated by dividing the weight (kg) by the squared height (m^2^), and obesity was defined as a BMI greater than 25 kg/m^2^ based on the Korean Society for the Study of Obesity [25]. Blood pressure, including systolic blood pressure (SBP) and diastolic blood pressure (DBP), was assessed using a mercury manometer in a sitting position.

Blood samples were collected after an overnight fast, and the fasting glucose (FG), triglycerides (TG), total cholesterol (TC), and high-density lipoprotein (HDL-C) levels were measured using a Hitachi automatic analyzer 7600 (Hitachi, Tokyo, Japan). Additionally, the concentration of 25 (OH) D with equimolar measurement of 25 (OH) D2 and 25 (OH) D3, which is a marker of the vitamin D status, was measured by radioimmunoassay kit (DiaSorin, Stillwater, MN, USA) using a 1470 WIZARD gamma-Counter (PerkinElmer, Turku, Finland). To improve the quality control for 25 (OH) D from the Korean National Health and Nutrition Examination Survey, the Korean Centers for Disease Control and Prevention (KCDC) continuously monitored and checked through internal and external quality control. The internal quality control, which is used through Westgard multi-rule control method, was evaluated the precision and accuracy of a analysis system by mean, standard deviation (SD) and coefficient of variation (CV%). And, external quality control was evaluated Vitamin D Quality Assessment Scheme (DEQAS), Vitamin D Metabolites Quality Assurance Program (VitDQAP) of National Institute of Standards and Technology (NIST), Accuracy-based Vitamin D survey of College of American Pathologists (CAP). The participants were divided into the following two categories according to their 25 (OH) D concentration based on the Institute of Medicine [26]: Vitamin D insufficient (<50 nmol/L) and sufficient (≥50 nmol/L).

### 2.3. Statistical Analyses

To best represent the entire Korean population, all statistical analyses used the sample weights provided by the KNHANES [26]. Continuous variables were examined for normal distribution, and skewed variables were performed logarithmic transformations, such as TC, HDL-C, and TG. According to the 25 (OH) D concentrations, the general characteristics were examined by performing Pearson’s chi-square test or independent *t*-tests. To avoid confounding effects, the socioeconomic variables (i.e., education level, monthly household income, and marriage status) and health-related variables (i.e., age, gender, subjective stress level, smoking status, drinking status, and physical activity) were included as covariates. After adjusting for the covariates, a general linear model was used to evaluate the differences in the obesity-related variables and dietary macronutrient consumption based on the 25 (OH) D concentration or sleep duration. The obesity-related variables and dietary macronutrient consumption was also examined based on the 25 (OH) D concentration and sleep duration using a general linear model after adjusting for the covariates. Multivariable logistic regression models after adjusting for the covariates were used to estimate the risk of obesity based on the 25 (OH) D concentration and sleep duration. After accounting for dietary macronutrient consumption, the risk of obesity based on the 25 (OH) D concentration and sleep duration was estimated using 25 (OH) D concentration ≥21.0 ng/mL and sleep duration ≥7 h/day as references in a multivariable logistic regression analysis after adjusting for the covariates. All statistical analyses were performed using SPSS software for Windows (version 21.0; IBM Corp., Armonk, NY, USA). A *p*-value <0.05 was considered statistically significant.

## 3. Results

### 3.1. General Characteristics

The general characteristics of the participants stratified according to the 25 (OH) D concentration are presented in Table 1. The average age and 25 (OH) D concentration were 71.61 years (65–97 years) and 47.58 nmol/L (10.28–133.85 nmol/L), respectively. The percentage of men in the 25 (OH) D sufficient and insufficient groups was 50.3% and 40.3%, respectively (*p* < 0.001). However, no differences were observed in age, education level, monthly household income, subjective stress, smoking status, and physical activity between the 25 (OH) D sufficient and insufficient groups; alcohol consumption (*p* = 0.006), living status (*p* < 0.001), and supplement consumption (*p* < 0.001) significantly differed between the groups

### 3.2. Obesity-Related Variables and Dietary Macronutrient Consumption by 25 (OH) D Concentration

The obesity-related variables and dietary macronutrient consumption were adjusted for age, gender, education level, monthly household income, subjective stress, smoking status, drinking status, and physical activity and are shown by the 25 (OH) D group in Table 2. The mean sleep duration in the 25 (OH) D sufficient and insufficient groups was 6.67 and 6.50 h, respectively (*p* = 0.005). The obesity-related anthropometric variables, including BMI (*p* = 0.001) and WC (*p* = 0.014), were significantly higher in the 25 (OH) D insufficient group than those in the sufficient group. The participants in the 25 (OH) D insufficient group had higher FG (*p* = 0.016) and TG (*p* = 0.019) than those in the 25 (OH) D sufficient group. However, no significant differences were observed in BP, HDL-C, and LDL-C between the 25 (OH) D sufficient and insufficient groups.

Dietary energy consumption was significantly associated with the 25 (OH) D concentration (*p* < 0.001), but the consumption of other macronutrients did not differ after adjusting for the covariates.

### 3.3. Effect of 25 (OH) D Concentration and Sleep Duration on Obesity and Dietary Macronutrient Consumption

**Figure 1:**
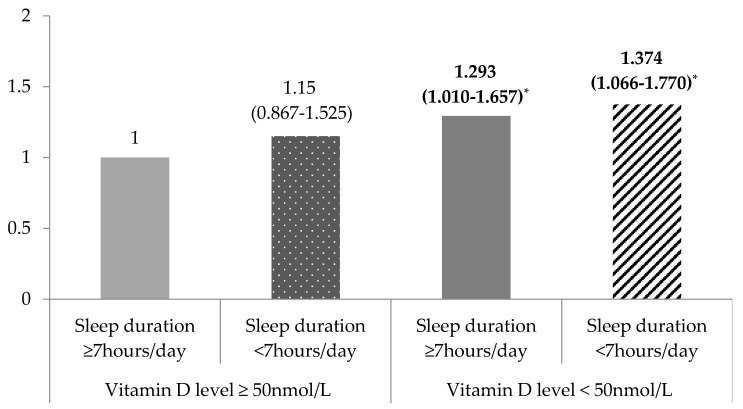
Adjusted odds ratio for obesity according to the 25-hydroxyvitamin D concentration and sleep duration. The odds ratios (95% confidence intervals) were calculated in reference to a serum vitamin D concentration ≥50 nmol/L and sleep duration ≥7 h using multivariate logistic regression after adjusting for age, gender, education level, monthly household income, marital status, subjective stress level, smoking status, drinking status, and physical activity (* *p* < 0.0001).

To determine whether 25 (OH) D concentration and sleep duration simultaneously affected obesity and dietary macronutrient consumption, a general linear model and a multivariate logistic regression model were analyzed after adjusting for the covariates (Table 3 and Figure 1). Significant differences were observed in the interaction effect of the 25 (OH) D concentration and sleep duration on BMI (*p* = 0.004). Participants who were 25 (OH) D insufficient and slept <7 h a day had higher BMI values than those who were 25 (OH) D sufficient and slept ≥7 h a day. Moreover, TG (*p* = 0.041) and dietary energy consumption (*p* < 0.001) were associated with 25 (OH) D concentration and sleep duration, but these associations did not demonstrate a particular trend. However, no significant differences were observed in WC, BP, FG, TC, HDL-C and dietary macronutrient consumption based on the 25 (OH) D concentration and sleep duration.

The adjusted odds ratios of obesity according to the 25 (OH) D concentration and sleep duration were significant (*p* < 0.001, Figure 1). Using the group with sufficient 25 (OH) D and a sleep duration ≥7 h a day as a reference, there was no difference in the risk of obesity in the 25 (OH) D sufficient group regardless of sleep duration. However, the risk of obesity significantly differed among the participants with insufficient 25 (OH) D. In other words, among the participants who were 25 (OH) D insufficient, the risk of obesity was significantly increased by 1.293 (95% CI = 1.010–1.657) for sleep duration ≥7 h a day and 1.374 times (95% CI = 1.066–1.770) for sleep duration <7 h a day.

### 3.4. Combined Effect of 25 (OH) D Concentration and Sleep Duration on Obesity Risk According to Dietary Macronutrient Consumption

After the participants were stratified into groups according to dietary macronutrient consumption, the effects of 25 (OH) D status and sleep duration on the risk of obesity were calculated using multivariate logistic regression models (Table 4). Among the participants with a low consumption of dietary protein, defined as less than the median value, compared to that of participants with sufficient 25 (OH) D concentrations and a sleep duration ≥7 h a day, the risk of obesity showed a significantly increasing trend in subjects with insufficient 25 (OH) D concentrations and a sleep duration <7 h a day. In other words, among the participants with low dietary protein consumption, the ORs of obesity were 1.590 times higher (95% CI = 1.041–2.430) for those with 25 (OH) D insufficiency and a sleep duration <7 h a day, 1.645 times higher (95% CI = 1.155–2.344) for those with insufficient 25 (OH) D and a sleep duration ≥7 h a day, and 1.668 times higher (95% CI = 1.156–2.406) than in those with sufficient 25 (OH) D and a sleep duration ≥7 h a day. However, no association was observed between the interaction of 25 (OH) D status and sleep duration in the risk of obesity among participants with high dietary protein consumption. Indeed, no significant differences in the risk of obesity were observed in the interaction between 25 (OH) D and sleep duration regardless of dietary energy, CHO, and fat consumption.

## 4. Discussion

Using data from the KNHANES, which is a nationally representative survey of the Korean population, this study examined whether dietary macronutrient consumption affects the risk of obesity in relation to the interaction between vitamin D status and sleep duration in participants older than 65 years. The obesity-related variables and dietary macronutrient consumption significantly differed between the groups with varying 25 (OH) D levels after adjusting for age, gender, education level, monthly household income, subjective stress, smoking status, drinking status, and physical activity. Additionally, the risk of obesity was influenced by the interaction between vitamin D status and sleep duration. Interestingly, this interaction was modified by dietary macronutrient consumption.

This study was consistent with previous studies [9,10,11,27] that have shown that the serum vitamin D concentration is associated with obesity-related variables, such as BMI, WC, FG, and TG, in elderly populations. In a recent meta-analysis, the prevalence of vitamin D deficiency was reported to be greater in overweight and obese individuals [10]. Additionally, an inverse association between increased adiposity and vitamin D concentrations has been observed in previous studies [9,27]. Specifically, Forsythe et al. reported that vitamin D concentrations were inversely associated with obesity-related variables, including BMI, WC, and fat mass, in older adults but not in younger adults [28]. Similarly, the relationship between obesity and vitamin D deficiency is well established. Although the mechanism responsible for the lower vitamin D concentrations in obese individuals is unclear, several possible mechanisms have been suggested: (1) lower consumption of vitamin D; (2) decreased dermal synthesis due to decreased sun exposure, synthetic capacity and intestinal absorption; and (3) altered metabolism, including decreased activation and/or increased catabolism of 25 (OH) D and the sequestration of 25 (OH) D within adipose tissue [11].

In our previous studies [16,20], I reported that a short sleep duration is associated with increased obesity. However, an association between sleep duration and obesity was not found in this study. These discrepancies might partly explain the sleep problems that are frequently reported by many older individuals [17], as mentioned in the Introduction. In other words, even if the sleep duration of the elderly is similar to that of different age groups, it may not affect obesity due to differences in sleep quality induced by the presence of sleep problems. However, the combined effect of vitamin D status and sleep duration on obesity was observed after adjusting for the covariates in this study. Compared with the same parameters in participants with sufficient vitamin D, BMI and risk of obesity in participants with insufficient vitamin D showed an increasing trend, particularly among participants with a short sleep duration. Several studies have demonstrated the associations between low 25 (OH) D concentrations and poor sleep patterns [29,30,31]. Bertisch et al. [29] reported that participants with vitamin D deficiency have shorter sleep durations, low sleep efficiency, and high sleepiness scores. Indeed, a significant improvement in sleep patterns, including longer sleep durations and better sleep quality (decreased sleep latency and reformed sleep efficiency), has been observed after 3 months of vitamin D supplementation [31]. Although an increase in obesity due to a short sleep duration was not found in the elderly population in the present study, the increased risk of obesity was affected by a short sleep duration in participants with vitamin D deficiency. The mechanism for the association between sleep duration and vitamin D status on obesity could not be fully explained. However, a plausible mechanism may be partly explained by the relationship between sleep duration and sleep quality in the elderly population. In other words, although a proper sleep duration has been reported to be associated with good sleep quality [18], differences in sleep quality regardless of sleep duration might be observed in older individuals. In particular, because vitamin D influences brain stem control of sleep [31], short sleep duration among participants with vitamin D deficiency might result in poor sleep quality and thus, could lead to an increase in obesity.

Among the participants with high dietary protein consumption, no difference was observed in the risk of obesity based on the combined effect of the vitamin D status and sleep duration. However, interestingly, participants with insufficient vitamin D and shorter sleep durations had a significantly higher risk of obesity than participants with sufficient vitamin D and longer sleep durations among the participants who consumed less dietary protein. As mentioned in the introduction, inadequate dietary consumption and exposure to sunlight could cause vitamin D deficiency [1,2,31]. The elderly have a decreased level of the precursor 7-dehydrocholesterol, which is found in the skin, and consequently have a decreased capacity to produce vitamin D. Additionally, the elderly consume less dietary protein, even though the main sources of vitamin D are mostly animal-based proteins, such as fatty fish, egg yolks, and dairy products. Furthermore, obesity in the elderly is associated with decreased outdoor activity and insufficient dietary protein consumption, which further increase the risk of vitamin D deficiency. Therefore, maintaining adequate vitamin D concentrations by increasing outdoor activity and protein consumption may prevent short sleep duration-induced obesity.

This study demonstrated that sleep durations may influence obesity among participants with vitamin D insufficiency and that this relationship is modified by dietary consumption. However, these findings have several limitations. First, the results of the present study could only estimate associations and not draw causal relationships due to the use of a cross-sectional study design. Second, the concentration of vitamin D is affected by several environmental and demographic factors. Although the vitamin D concentration was adjusted for several available factors, season of the blood sampling, sun exposure and use of a vitamin D supplement could not be assessed. Also, radioimmunoassay, a method for measurement of vitamin D, is widely used, however this method has limited accuracy and lack sufficient standardization [32]. Future studies should adjust for more detailed factors affecting the vitamin D concentration and need to examine more sensitive method for vitamin D status. Third, the sleep duration variable, which was assessed using a self-reported questionnaire, considered sleep problems because a proper sleep duration is associated with good sleep quality [18], therefore, both objective and accurate sleep duration and quality should be investigated simultaneously. Finally, the definition of obesity in this study was as a BMI greater than 25 kg/m^2^, which is a low cutoff point compared to that of western countries. Therefore, these results may not be fully generalizable to other populations, and future studies are required to confirm these results in different populations.

## 5. Conclusions

Using representative data of the elderly Korean population from the KNHANES, this study demonstrated that the vitamin D concentration is related to differences in obesity-related variables. Indeed, a short sleep duration was associated with an increased risk of obesity among participants with vitamin D deficiency, and the risk of obesity increased with low dietary protein consumption. Thus, the increased risk of obesity due to short sleep durations among vitamin D deficient populations is likely modulated by dietary protein consumption.

## Figures and Tables

**Table 1:** General characteristics according to the 25-hydroxyvitamin D concentration.

	Sufficient ≥50 nmol/L (*n* = 1515)	Insufficient <50 nmol/L (*n* = 2242)	*p*-Value *
Gender, men	50.3	40.3	<0.001
Age, years	71.63 ± 0.17	71.60 ± 0.14	0.871
Education level, ≥high school	20.3	21.2	0.923
Living together	70.8	64.7	<0.001
Income, 10,000 won/month	253.41 ± 40.32	207.56 ± 9.67	0.268
Subjective stress, high	18.4	21.6	0.051
Regular alcohol drinker	40.5	34.6	0.006
Current smoker	12.8	13.2	0.745
Supplement consumption, yes	50.9	42.5	<0.001
Regular physical activity	46.9	46.2	0.718

The data are expressed as the means ± SEM or %; * *p*-values between sleep duration using x^2^-test or *t*-test.

**Table 2:** Obesity-related variables and dietary macronutrient consumption according to the 25-hydroxyvitamin D concentration.

	Sufficient ≥50 nmol/L (*n* = 1515)	Insufficient <50 nmol/L (*n* = 2242)	*p*-Value *
Sleep duration (h)	6.67 ± 0.05	6.50 ± 0.04	0.005
BMI (kg/m^2^)	23.65 ± 0.10	24.08 ± 0.09	0.001
WC (cm)	83.59 ± 0.36	84.60 ± 0.26	0.014
SBP (mmHg)	130.25 ± 0.63	130.89 ± 0.49	0.393
DBP (mmHg)	74.78 ± 0.34	74.60 ± 0.29	0.683
FG (mg/dL)	102.49 ± 0.64	104.68 ± 0.67	0.016
TG (mg/dL)	136.12 ± 2.76	144.81 ± 2.50	0.019
TC (mg/dL)	190.47 ± 1.22	190.20 ± 0.99	0.863
HDL-C (mg/dL)	52.32 ± 0.40	52.35 ± 0.35	0.947
LDL-C (mg/dL)	111.72 ± 1.20	109.61 ± 0.97	0.171
Dietary consumption			
Energy (kcal)	1742.42 ± 19.13	1655.51 ± 15.56	<0.001
%_CHO	74.00 ± 0.37	74.28 ± 0.28	0.743
%_PRO	12.73 ± 0.12	12.63 ± 0.10	0.501
%_FAT	13.27 ± 0.33	13.15 ± 0.25	0.759

The data are expressed as the means ± SEM; BMI, body mass index; WC, waist circumference; SBP, systolic blood pressure; DBP, diastolic blood pressure; FG, fasting glucose; TG, triglycerides; TC, total cholesterol; HDL-C, high-density lipoprotein cholesterol; LDL-C, low-density lipoprotein cholesterol; * *p*-values between serum vitamin D concentration using a general linear model after adjusting for age, gender, education level, monthly household income, marital status, subjective stress level, smoking status, drinking status, and physical activity.

**Table 3:** Obesity-related variables and dietary macronutrient consumption according to the 25-hydroxyvitamin D concentration and sleep duration.

	Sufficient ≥50 nmol/L		Insufficient <50 nmol/L		*p*-Value *
	≥7 h/day (*n* = 833)	<7 h/day (*n* = 682)	≥7 h/day (*n* = 833)	<7 h/day (*n* = 682)	
BMI (kg/m^2^)	23.52 ± 0.13	23.81 ± 0.15	24.01 ± 0.11	24.14 ± 0.13	0.004
WC (cm)	83.47 ± 0.41	83.75 ± 0.46	84.35 ± 0.34	84.83 ± 0.35	0.063
SBP (mmHg)	130.45 ± 0.82	130.01 ± 0.82	130.78 ± 0.67	130.99 ± 0.64	0.073
DBP (mmHg)	75.01 ± 0.44	74.50 ± 0.48	74.34 ± 0.38	74.86 ± 0.36	0.544
FG (mg/dL)	102.05 ± 0.81	103.04 ± 1.00	105.21 ± 0.98	104.16 ± 0.88	0.075
TG (mg/dL)	138.63 ± 4.18	133.23 ± 3.84	147.86 ± 3.49	141.82 ± 3.60	0.041
TC (mg/dL)	191.21 ± 1.65	189.60 ± 1.71	190.08 ± 1.40	190.30 ± 1.40	0.915
HDL-C (mg/dL)	52.45 ± 0.56	52.16 ± 0.55	51.93 ± 0.46	52.75 ± 0.53	0.682
Dietary consumption					
Energy (kcal)	1795.42 ± 23.36	1681.40 ± 28.13	1626.95 ± 21.46	1681.69 ± 20.60	<0.001
%_CHO	73.79 ± 0.51	74.25 ± 0.49	74.65 ± 0.40	73.83 ± 0.37	0.364
%_PRO	12.74 ± 0.16	12.73 ± 0.17	12.72 ± 0.13	12.53 ± 0.13	0.6
%_FAT	13.48 ± 0.44	13.03 ± 0.44	12.63 ± 0.34	13.64 ± 0.34	0.138

The data are expressed as the means ± SEM; BMI, body mass index; WC, waist circumference; SBP, systolic blood pressure; DBP, diastolic blood pressure; FG, fasting glucose; TG, triglycerides; TC, total cholesterol; HDL-C, high-density lipoprotein cholesterol; LDL-C, low-density lipoprotein cholesterol; * p-values for the interaction between sleep duration and serum vitamin D using a general linear model after adjusting for age, gender, education level, monthly household income, marital status, subjective stress level, smoking status, drinking status, and physical activity.

**Table 4:** Association between the 25-hydroxyvitamin D concentration and sleep duration with obesity according to dietary macronutrient consumption.

		Vitamin D ≥50 nmol/L		Vitamin D <50 nmol/L		
		Sleep Duration ≥7 h	Sleep Duration <7 h	Sleep Duration ≥7 h	Sleep Duration <7 h	
Energy	Low	Reference	1.398	1.399	1.473	0.129
			(0.899–2.174)	(0.936–2.091)	(0.989–2.193)	
	High	Reference	0.958	1.22	1.339	0.231
			(0.676–1.359)	(0.874–1.702)	(0.943–1.900)	
CHO	Low	Reference	0.949	1.216	1.268	0.333
			(0.646–1.393)	(0.854–1.731)	(0.888–1.811)	
	High	Reference	1.43	1.41	1.522	0.109
			(0.933–2.192)	(0.990–2.008)	(1.080–2.146)	
Protein	Low	Reference	1.59	1.645	1.668	0.201
			(1.041–2.430) *	(1.155–2.344) *	(1.156–2.406) *	
	High	Reference	0.858	1.066	1.187	0.316
			(0.576–1.278)	(0.740–1.535)	(0.831–1.696)	
Fat	Low	Reference	1.357	1.244	1.372	0.345
			(0.883–2.085)	(0.873–1.774)	(0.964–1.954)	
	High	Reference	0.971	1.351	1.374	0.999
			(0.650–1.450)	(0.957–1.907)	(0.970–1.945)	

The data are expressed as the odds ratio (ORs) and 95% confidence intervals (CIs). ORs (95% CIs) were calculated in reference to a serum vitamin D concentration ≥21.0 ng/mL and sleep duration ≥7 h/day using multivariate logistic regression after adjusting for age, gender, education level, monthly household income, marital status, subjective stress level, smoking status, drinking status, and physical activity (* *p* < 0.0001).
